# The London Polyclinic

**Published:** 1907-09-07

**Authors:** 


### THE LONDON POLYCLINIC.

The Medical Graduates' College and Polyclinic, to quote its full title, has now a well-recognised position among the medical educational organisations of the metropolis. As is implied by its name, its object is to provide opportunities for clinical and practical study for those who have already gained a position on the medical register. From the outset, two principles have been recognised as essential to a large and broad scheme of post-graduation study. First, that such a scheme should be entirely separate and distinct from the ordinary educational course of the medical student. Secondly, that the teaching should not be confined to the staff of any individual hospital, but should include capable and distinguished representatives of all schools. Hence, at the Polyclinic, the needs of the practitioner receive full consideration, and the teaching in a very special manner represents the work of many of the best-known physicians and surgeons in the metropolis. It is this broad and comprehensive platform which makes the institution one of peculiar value to those anxious to come into contact with the latest and best work of the day.

Coming now to details, there is first to be noted the afternoon cliniques. These are conducted daily, and they offer very exceptional and valuable opportunities. Medicine, surgery, and each of the more special branches of practice, has its appropriate day. Selected cases are demonstrated and made the subject of clinical comment, and opportunities for personal examination by those attending the clinique are offered. In this way, and in a comparatively short period of time, the practitioner is able to overtake a large quantity of interesting and instructive clinical material under the direction of teachers specially qualified in each of the various departments. Indeed, it is difficult to imagine any scheme more suitable for those who are anxious to increase their clinical experience both in general and special work. On four days of each week there is also a lecture, and here teachers from the provincial as well as from the metropolitan schools take part in the programme. The range of subjects may be said to cover the entire professional field, and in many instances the "lecture" assumes the form of a lantern demonstration or of a discussion of selected pathological or clinical specimens. Thus the practical note is maintained throughout. With a view to render these opportunities accessible to all, the annual subscription, which includes admission to all cliniques and lectures and also the use of the library and museum, has been fixed at one guinea. In addition, the *Polyclinic Journal* is issued monthly, and is sent free to all subscribers; and in the college laboratory specimens are examined and reported on for small fees. Another part of the Polyclinic scheme, and one of great importance, is the provision of "practical classes." It is recognised that many practitioners have not had adequate opportunities for equipping themselves with the more recent methods of clinical investigation, and an attempt is therefore made to meet this want in the classes to which reference is now made. Included in this division, instruction is provided in the use of the ophthalmoscope, laryngoscope, otoscope, and other specialised clinical instruments; in the clinical examination of the blood, sputa, urine, etc.; in the use of the *x*-rays; and in practical gynæcology. In each class the numbers are limited, so that each member is able to receive the direct personal aid of the teacher, and patients are examined under the most favourable conditions. A special vacation course of these practical classes is to commence on Monday, September 9th, and is completed by September 27.

The success already achieved by the Polyclinic is a considerable one, and it appears to have a future of still greater usefulness before it. In providing practical and clinical opportunities for practitioners, and especially for those whose time for study is limited, it serves a highly useful purpose, and, indeed, it is now a matter for wonder that such an institution was not founded at a much earlier date. Doubtless time will lead to change and to development, and to new demands the Polyclinic will probably be able readily to respond. At present it certainly discharges a function of great value, not only to the profession, but also to the public, and we regard it as worthy of cordial and energetic support.

